# Supplementary material for: Age-specific trends in limitations of daily activities in American adults aged 50–84 by race and ethnicity, 2000–2018
Source: PLoS One. 2026 Feb 23;21(2):e0340694. doi: 10.1371/journal.pone.0340694 (PMC12928396; doi:10.1371/journal.pone.0340694)
Supplement: S1 Table — (DOCX) [file pone.0340694.s001.docx]

**Table 1S.** Foreign-born and US-born observations among complete cases by race-ethnicity.

| **Race-Ethnicity** | **Foreign-born** | **US-born** | **N/A (dropped)** | **Total cases (TC)** | **Complete Cases (CC)** | **% Foreign-born on CC** | **% N/A on TC** |
| --- | --- | --- | --- | --- | --- | --- | --- |
| NH-White | 17,442 | 333,098 | 1,100 | 351,640 | 350,540 | 4.97 (0.04) | 0.31 (0.0006) |
| NH-Black | 6,067 | 56,389 | 238 | 62,694 | 62,456 | 9.71 (0.04) | 0.37 (0.0006  ) |
| Hispanic | 38,773 | 25,402 | 359 | 64,534 | 64,175 | 60.41 (0.08) | 0.55 (0.0012) |
