# Supplementary material for: Age-specific trends in limitations of daily activities in American adults aged 50–84 by race and ethnicity, 2000–2018
Source: PLoS One. 2026 Feb 23;21(2):e0340694. doi: 10.1371/journal.pone.0340694 (PMC12928396; doi:10.1371/journal.pone.0340694)
Supplement: S2 Table — (DOCX) [file pone.0340694.s002.docx]

**Table 2S.** Logistic regression results for all 12 models, stratified by age and sex

| **Term** | **Coefficient** | **Std. Error** | **Z-statistic** | **P-value** | **Sig.** |
| --- | --- | --- | --- | --- | --- |
| **Female 50-64 ADL** | | | | | |
| (Intercept) | -5.374 | 0.332 | -16.189 | <0.001 | *** |
| Year | 0.041 | 0.011 | 3.804 | <0.001 | *** |
| Year After 2010 | -0.032 | 0.022 | -1.445 | 0.1486 |  |
| Non-Hispanic Black | 0.553 | 0.115 | 4.791 | <0.001 | *** |
| Hispanic US Born | 0.221 | 0.165 | 1.336 | 0.1819 |  |
| Hispanic Foreign Born | -1.067 | 0.179 | -5.943 | <0.001 | *** |
| Age | 0.039 | 0.006 | 6.986 | <0.001 | *** |
| GED to Some College | -0.821 | 0.059 | -13.991 | <0.001 | *** |
| Bachelor’s Degree and Beyond | -1.588 | 0.077 | -20.733 | <0.001 | *** |
| Year *Non-Hispanic Black | 0.008 | 0.022 | 0.358 | 0.7204 |  |
| Year *Hispanic US-born | 0.012 | 0.030 | 0.391 | 0.6958 |  |
| Year *Hispanic Foreign Born | -0.079 | 0.032 | -2.464 | 0.0139 | * |
| Year After 2010* Non-Hispanic Black | -0.025 | 0.045 | -0.562 | 0.5745 |  |
| Year After 2010* Hispanic US-born | 0.012 | 0.060 | 0.204 | 0.8381 |  |
| Year After 2010* Hispanic Foreign-born | 0.156 | 0.064 | 2.436 | 0.015 | * |

| **Female 50-64 IADL** | | | | | |
| --- | --- | --- | --- | --- | --- |
| (Intercept) | -8.185 | 0.722 | -11.335 | <0.001 | *** |
| Year | -0.003 | 0.013 | -0.224 | 0.8225 |  |
| Year After 2010 | 0.028 | 0.026 | 1.069 | 0.2853 |  |
| Non-Hispanic Black | 0.514 | 0.156 | 3.306 | 0.001 | ** |
| Hispanic US Born | 0.156 | 0.238 | 0.655 | 0.5125 |  |
| Hispanic Foreign Born | 0.209 | 0.210 | 0.996 | 0.3192 |  |
| Age | 0.071 | 0.010 | 6.931 | <0.001 | *** |
| GED to Some College | -0.371 | 0.070 | -5.302 | <0.001 | *** |
| Bachelor’s Degree and Beyond | -0.736 | 0.091 | -8.086 | <0.001 | *** |
| Year *Non-Hispanic Black | -0.004 | 0.028 | -0.133 | 0.8945 |  |
| Year *Hispanic US-born | -0.033 | 0.039 | -0.852 | 0.3946 |  |
| Year *Hispanic Foreign Born | 0.037 | 0.038 | 0.991 | 0.322 |  |
| Year After 2010* Non-Hispanic Black | 0.002 | 0.057 | 0.042 | 0.9663 |  |
| Year After 2010* Hispanic US-born | 0.102 | 0.082 | 1.232 | 0.2182 |  |
| Year After 2010* Hispanic Foreign-born | -0.049 | 0.078 | -0.620 | 0.5351 |  |

| **Female 65-74 ADL** | | | | | |
| --- | --- | --- | --- | --- | --- |
| (Intercept) | -10.664 | 0.789 | -13.512 | <0.001 | *** |
| Year | -0.014 | 0.011 | -1.243 | 0.2139 |  |
| Year After 2010 | 0.033 | 0.024 | 1.391 | 0.1644 |  |
| Non-Hispanic Black | 0.543 | 0.162 | 3.346 | <0.001 | *** |
| Hispanic US Born | 0.434 | 0.268 | 1.620 | 0.1055 |  |
| Hispanic Foreign Born | 0.541 | 0.204 | 2.652 | 0.0081 | ** |
| Age | 0.101 | 0.010 | 10.317 | <0.001 | *** |
| GED to Some College | -0.271 | 0.065 | -4.137 | <0.001 | *** |
| Bachelor’s Degree and Beyond | -0.440 | 0.082 | -5.380 | <0.001 | *** |
| Year *Non-Hispanic Black | 0.037 | 0.032 | 1.153 | 0.2493 |  |
| Year *Hispanic US-born | 0.021 | 0.048 | 0.441 | 0.6595 |  |
| Year *Hispanic Foreign Born | 0.007 | 0.045 | 0.158 | 0.8746 |  |
| Year After 2010* Non-Hispanic Black | -0.056 | 0.063 | -0.886 | 0.3756 |  |
| Year After 2010* Hispanic US-born | -0.037 | 0.105 | -0.353 | 0.724 |  |
| Year After 2010* Hispanic Foreign-born | -0.059 | 0.092 | -0.643 | 0.5203 |  |

| **Female 65-74 IADL** | | | | | |
| --- | --- | --- | --- | --- | --- |
| (Intercept) | -5.234 | 0.289 | -18.133 | <0.001 | *** |
| Year | 0.042 | 0.010 | 4.201 | <0.001 | *** |
| Year After 2010 | -0.039 | 0.020 | -1.959 | 0.0503 | † |
| Non-Hispanic Black | 0.610 | 0.098 | 6.245 | <0.001 | *** |
| Hispanic US Born | 0.213 | 0.135 | 1.584 | 0.1134 |  |
| Hispanic Foreign Born | -0.845 | 0.153 | -5.521 | <0.001 | *** |
| Age | 0.040 | 0.005 | 8.450 | <0.001 | *** |
| GED to Some College | -0.819 | 0.054 | -15.306 | <0.001 | *** |
| Bachelor’s Degree and Beyond | -1.477 | 0.073 | -20.143 | <0.001 | *** |
| Year *Non-Hispanic Black | 0.015 | 0.017 | 0.903 | 0.3667 |  |
| Year *Hispanic US-born | -0.014 | 0.025 | -0.547 | 0.5842 |  |
| Year *Hispanic Foreign Born | -0.050 | 0.026 | -1.915 | 0.0557 | † |
| Year After 2010* Non-Hispanic Black | -0.016 | 0.034 | -0.468 | 0.6402 |  |
| Year After 2010* Hispanic US-born | 0.083 | 0.049 | 1.679 | 0.0933 | † |
| Year After 2010* Hispanic Foreign-born | 0.117 | 0.058 | 2.015 | 0.0442 | * |

| **Female 75-84 ADL** | | | | | |
| --- | --- | --- | --- | --- | --- |
| (Intercept) | -7.401 | 0.539 | -13.722 | <0.001 | *** |
| Year | 0.034 | 0.011 | 3.009 | 0.0027 | ** |
| Year After 2010 | -0.046 | 0.022 | -2.084 | 0.0373 | * |
| Non-Hispanic Black | 0.588 | 0.125 | 4.726 | <0.001 | *** |
| Hispanic US Born | 0.260 | 0.221 | 1.176 | 0.2396 |  |
| Hispanic Foreign Born | 0.118 | 0.175 | 0.678 | 0.4979 |  |
| Age | 0.068 | 0.008 | 8.813 | <0.001 | *** |
| GED to Some College | -0.668 | 0.057 | -11.817 | <0.001 | *** |
| Bachelor’s Degree and Beyond | -0.942 | 0.079 | -11.988 | <0.001 | *** |
| Year *Non-Hispanic Black | -0.017 | 0.021 | -0.800 | 0.4236 |  |
| Year *Hispanic US-born | -0.022 | 0.036 | -0.607 | 0.5439 |  |
| Year *Hispanic Foreign Born | -0.020 | 0.030 | -0.662 | 0.5083 |  |
| Year After 2010* Non-Hispanic Black | 0.071 | 0.043 | 1.638 | 0.1016 |  |
| Year After 2010* Hispanic US-born | 0.117 | 0.079 | 1.480 | 0.1392 |  |
| Year After 2010* Hispanic Foreign-born | 0.033 | 0.060 | 0.550 | 0.5825 |  |

| **Female 75-84 IADL** | | | | | |
| --- | --- | --- | --- | --- | --- |
| (Intercept) | -10.484 | 0.558 | -18.782 | <0.001 | *** |
| Year | 0.008 | 0.009 | 0.838 | 0.4023 |  |
| Year After 2010 | -0.001 | 0.019 | -0.029 | 0.9771 |  |
| Non-Hispanic Black | 0.789 | 0.106 | 7.410 | <0.001 | *** |
| Hispanic US Born | 0.730 | 0.162 | 4.517 | <0.001 | *** |
| Hispanic Foreign Born | 0.706 | 0.153 | 4.622 | <0.001 | *** |
| Age | 0.103 | 0.007 | 14.894 | <0.001 | *** |
| GED to Some College | -0.413 | 0.047 | -8.815 | <0.001 | *** |
| Bachelor’s Degree and Beyond | -0.326 | 0.064 | -5.131 | <0.001 | *** |
| Year *Non-Hispanic Black | 0.002 | 0.018 | 0.110 | 0.9121 |  |
| Year *Hispanic US-born | 0.010 | 0.029 | 0.333 | 0.7391 |  |
| Year *Hispanic Foreign Born | 0.044 | 0.027 | 1.637 | 0.1018 |  |
| Year After 2010* Non-Hispanic Black | -0.003 | 0.040 | -0.074 | 0.9413 |  |
| Year After 2010* Hispanic US-born | -0.022 | 0.062 | -0.363 | 0.7165 |  |
| Year After 2010* Hispanic Foreign-born | -0.011 | 0.055 | -0.193 | 0.8473 |  |

| **Male 50-64 ADL** | | | | | |
| --- | --- | --- | --- | --- | --- |
| (Intercept) | -4.342 | 0.254 | -17.067 | <0.001 | *** |
| Year | 0.029 | 0.008 | 3.734 | <0.001 | *** |
| Year After 2010 | -0.024 | 0.016 | -1.499 | 0.1342 |  |
| Non-Hispanic Black | 0.458 | 0.094 | 4.893 | <0.001 | *** |
| Hispanic US Born | 0.180 | 0.132 | 1.367 | 0.1719 |  |
| Hispanic Foreign Born | -1.210 | 0.159 | -7.609 | <0.001 | *** |
| Age | 0.032 | 0.004 | 7.678 | <0.001 | *** |
| GED to Some College | -0.842 | 0.046 | -18.341 | <0.001 | *** |
| Bachelor’s Degree and Beyond | -1.629 | 0.058 | -28.053 | <0.001 | *** |
| Year *Non-Hispanic Black | 0.012 | 0.017 | 0.670 | 0.5032 |  |
| Year *Hispanic US-born | -0.006 | 0.023 | -0.258 | 0.7964 |  |
| Year *Hispanic Foreign Born | -0.047 | 0.029 | -1.632 | 0.1029 |  |
| Year After 2010* Non-Hispanic Black | 0.000 | 0.034 | -0.012 | 0.9901 |  |
| Year After 2010* Hispanic US-born | 0.033 | 0.048 | 0.690 | 0.4901 |  |
| Year After 2010* Hispanic Foreign-born | 0.093 | 0.057 | 1.623 | 0.1049 |  |

| **Male 50-64 IADL** | | | | | |
| --- | --- | --- | --- | --- | --- |
| (Intercept) | -6.360 | 0.536 | -11.865 | <0.001 | *** |
| Year | -0.010 | 0.011 | -0.903 | 0.3668 |  |
| Year After 2010 | 0.032 | 0.021 | 1.542 | 0.1233 |  |
| Non-Hispanic Black | 0.564 | 0.127 | 4.443 | <0.001 | *** |
| Hispanic US Born | 0.355 | 0.175 | 2.030 | 0.0426 | * |
| Hispanic Foreign Born | 0.034 | 0.183 | 0.185 | 0.8533 |  |
| Age | 0.054 | 0.008 | 7.109 | <0.001 | *** |
| GED to Some College | -0.579 | 0.057 | -10.140 | <0.001 | *** |
| Bachelor’s Degree and Beyond | -0.946 | 0.072 | -13.049 | <0.001 | *** |
| Year *Non-Hispanic Black | 0.026 | 0.023 | 1.176 | 0.24 |  |
| Year *Hispanic US-born | 0.015 | 0.032 | 0.476 | 0.6341 |  |
| Year *Hispanic Foreign Born | 0.039 | 0.032 | 1.223 | 0.2217 |  |
| Year After 2010* Non-Hispanic Black | -0.034 | 0.047 | -0.727 | 0.4673 |  |
| Year After 2010* Hispanic US-born | 0.007 | 0.062 | 0.105 | 0.9163 |  |
| Year After 2010* Hispanic Foreign-born | -0.052 | 0.069 | -0.761 | 0.4466 |  |

| **Male 65-74 ADL** | | | | | |
| --- | --- | --- | --- | --- | --- |
| (Intercept) | -11.057 | 0.637 | -17.370 | <0.001 | *** |
| Year | -0.018 | 0.009 | -2.037 | 0.0419 | * |
| Year After 2010 | 0.038 | 0.019 | 2.021 | 0.0434 | * |
| Non-Hispanic Black | 0.573 | 0.129 | 4.455 | <0.001 | *** |
| Hispanic US Born | 0.154 | 0.203 | 0.757 | 0.4493 |  |
| Hispanic Foreign Born | 0.379 | 0.201 | 1.882 | 0.0601 | † |
| Age | 0.115 | 0.008 | 14.513 | <0.001 | *** |
| GED to Some College | -0.463 | 0.056 | -8.230 | <0.001 | *** |
| Bachelor’s Degree and Beyond | -0.640 | 0.066 | -9.767 | <0.001 | *** |
| Year *Non-Hispanic Black | 0.012 | 0.023 | 0.527 | 0.598 |  |
| Year *Hispanic US-born | -0.010 | 0.036 | -0.269 | 0.7879 |  |
| Year *Hispanic Foreign Born | 0.024 | 0.033 | 0.748 | 0.4548 |  |
| Year After 2010* Non-Hispanic Black | -0.044 | 0.051 | -0.863 | 0.3881 |  |
| Year After 2010* Hispanic US-born | -0.001 | 0.083 | -0.016 | 0.9876 |  |
| Year After 2010* Hispanic Foreign-born | -0.080 | 0.078 | -1.034 | 0.3014 |  |

| **Male 65-74 IADL** | | | | | |
| --- | --- | --- | --- | --- | --- |
| (Intercept) | -3.945 | 0.199 | -19.812 | <0.001 | *** |
| Year | 0.026 | 0.007 | 3.921 | <0.001 | *** |
| Year After 2010 | -0.023 | 0.013 | -1.759 | 0.0788 | † |
| Non-Hispanic Black | 0.463 | 0.073 | 6.324 | <0.001 | *** |
| Hispanic US Born | 0.077 | 0.092 | 0.837 | 0.4026 |  |
| Hispanic Foreign Born | -0.926 | 0.117 | -7.937 | <0.001 | *** |
| Age | 0.031 | 0.003 | 9.487 | <0.001 | *** |
| GED to Some College | -0.755 | 0.037 | -20.182 | <0.001 | *** |
| Bachelor’s Degree and Beyond | -1.541 | 0.050 | -31.030 | <0.001 | *** |
| Year *Non-Hispanic Black | 0.002 | 0.013 | 0.157 | 0.8751 |  |
| Year *Hispanic US-born | -0.007 | 0.016 | -0.447 | 0.6548 |  |
| Year *Hispanic Foreign Born | -0.033 | 0.021 | -1.571 | 0.1164 |  |
| Year After 2010* Non-Hispanic Black | -0.023 | 0.026 | -0.864 | 0.3878 |  |
| Year After 2010* Hispanic US-born | 0.052 | 0.034 | 1.526 | 0.1273 |  |
| Year After 2010* Hispanic Foreign-born | 0.081 | 0.041 | 1.987 | 0.0472 | * |

| **Male 75-84 ADL** | | | | | |
| --- | --- | --- | --- | --- | --- |
| (Intercept) | -6.281 | 0.402 | -15.628 | <0.001 | *** |
| Year | 0.013 | 0.008 | 1.676 | 0.0939 | † |
| Year After 2010 | -0.025 | 0.016 | -1.530 | 0.1262 |  |
| Non-Hispanic Black | 0.508 | 0.089 | 5.720 | <0.001 | *** |
| Hispanic US Born | 0.442 | 0.144 | 3.079 | 0.0021 | ** |
| Hispanic Foreign Born | -0.074 | 0.131 | -0.570 | 0.5689 |  |
| Age | 0.063 | 0.006 | 11.012 | <0.001 | *** |
| GED to Some College | -0.670 | 0.042 | -15.841 | <0.001 | *** |
| Bachelor’s Degree and Beyond | -1.003 | 0.057 | -17.579 | <0.001 | *** |
| Year *Non-Hispanic Black | -0.010 | 0.016 | -0.622 | 0.5342 |  |
| Year *Hispanic US-born | 0.035 | 0.024 | 1.431 | 0.1528 |  |
| Year *Hispanic Foreign Born | 0.007 | 0.023 | 0.308 | 0.7584 |  |
| Year After 2010* Non-Hispanic Black | 0.028 | 0.032 | 0.877 | 0.3807 |  |
| Year After 2010* Hispanic US-born | -0.016 | 0.053 | -0.302 | 0.7625 |  |
| Year After 2010* Hispanic Foreign-born | 0.018 | 0.047 | 0.388 | 0.6979 |  |

| **Male 75-84 IADL** | | | | | |
| --- | --- | --- | --- | --- | --- |
| (Intercept) | -9.835 | 0.420 | -23.443 | <0.001 | *** |
| Year | -0.010 | 0.007 | -1.482 | 0.1386 |  |
| Year After 2010 | 0.005 | 0.014 | 0.359 | 0.7198 |  |
| Non-Hispanic Black | 0.563 | 0.087 | 6.482 | <0.001 | *** |
| Hispanic US Born | 0.425 | 0.142 | 2.987 | 0.0029 | ** |
| Hispanic Foreign Born | 0.292 | 0.131 | 2.230 | 0.026 | * |
| Age | 0.107 | 0.005 | 20.674 | <0.001 | *** |
| GED to Some College | -0.476 | 0.035 | -13.585 | <0.001 | *** |
| Bachelor’s Degree and Beyond | -0.490 | 0.050 | -9.724 | <0.001 | *** |
| Year *Non-Hispanic Black | -0.001 | 0.015 | -0.064 | 0.9492 |  |
| Year *Hispanic US-born | 0.020 | 0.023 | 0.886 | 0.3756 |  |
| Year *Hispanic Foreign Born | 0.054 | 0.021 | 2.519 | 0.0119 | * |
| Year After 2010* Non-Hispanic Black | -0.005 | 0.032 | -0.139 | 0.8898 |  |
| Year After 2010* Hispanic US-born | -0.014 | 0.052 | -0.262 | 0.7933 |  |
| Year After 2010* Hispanic Foreign-born | -0.034 | 0.047 | -0.718 | 0.4729 |  |

Significance codes: *** p<0.001, ** p<0.01, * p<0.05, † p<0.10. Note: Reference categories were Year 2000, Non-Hispanic Whites, First age for each age- group (50,65,74), Less than GED or High School Diploma.
