# Supplementary material for: Age-specific trends in limitations of daily activities in American adults aged 50–84 by race and ethnicity, 2000–2018
Source: PLoS One. 2026 Feb 23;21(2):e0340694. doi: 10.1371/journal.pone.0340694 (PMC12928396; doi:10.1371/journal.pone.0340694)
Supplement: S3 Table — (DOCX) [file pone.0340694.s003.docx]

**Table 3S.** Goodness of fit R-square tests for all 12 models

| **Model** |  | **McFadden R-square** |  | **Nagelkerke R-square** |  |  | **Pseudo R-Square** |
| --- | --- | --- | --- | --- | --- | --- | --- |
| **Male 50-64 ADL** | | 0.036316 |  | 0.039461 |  |  | 0.036316 |
| **Male 65-74 ADL** | | 0.018912 |  | 0.021487 |  |  | 0.018912 |
| **Male 75-84 ADL** | | 0.020159 |  | 0.024945 |  |  | 0.020159 |
| **Female 50-64 ADL** | | 0.035642 |  | 0.039397 |  |  | 0.035642 |
| **Female 65-74 ADL** | | 0.032483 |  | 0.037808 |  |  | 0.032483 |
| **Female 75-84 ADL** | | 0.033438 |  | 0.043484 |  |  | 0.033438 |
| **Male 50-64 IADL** | | 0.041155 |  | 0.046982 |  |  | 0.041155 |
| **Male 65-74 IADL** | | 0.022826 |  | 0.027381 |  |  | 0.022826 |
| **Male 75-84 IADL** | | 0.028853 |  | 0.038724 |  |  | 0.028853 |
| **Female 50-64 IADL** | | 0.034923 |  | 0.041822 |  |  | 0.034923 |
| **Female 65-74 IADL** | | 0.029741 |  | 0.038222 |  |  | 0.029741 |
| **Female 75-84 IADL** | | 0.029728 |  | 0.044599 |  |  | 0.029728 |
