# Supplementary material for: Age-specific trends in limitations of daily activities in American adults aged 50–84 by race and ethnicity, 2000–2018
Source: PLoS One. 2026 Feb 23;21(2):e0340694. doi: 10.1371/journal.pone.0340694 (PMC12928396; doi:10.1371/journal.pone.0340694)
Supplement: S4 Table — (DOCX) [file pone.0340694.s004.docx]

**Table S4.** Coefficients that changed p-value after False Discovery Rate (FDR) corrections using Benjamini-Hochberg procedure

Note: * is for coefficients that were significant (at p-value 0.05) before FDR correction but not after.

| **Model** | **Term** | **Estimate** | **Std. Error** | **Z-statistic** | **P-value (original)** | **P-value (FDR)** |  |  |
| --- | --- | --- | --- | --- | --- | --- | --- | --- |
| **Female 65-74 IADL** | | | | | | | | |
| Year After 2010 | | -0.039 | 0.020 | -1.959 | 0.0503 | 0.1065 |  |  |
| Year After 2010*Hispanic US-born | | 0.083 | 0.049 | 1.679 | 0.0933 | 0.1878 |  |  |
| Year*Hispanic Foreign-born | | -0.050 | 0.026 | -1.915 | 0.0557 | 0.1166 |  |  |
| **Male 65-74 ADL** | | | | | | | | |
| Hispanic Foreign-born | | 0.379 | 0.201 | 1.882 | 0.0601 | 0.1243 |  |  |
| **Male 65-74 IADL** | | | | | | | | |
| Year After 2010 | | -0.023 | 0.013 | -1.759 | 0.0788 | 0.1612 |  |  |
| Year After 2010*Hispanic Foreign Born | | 0.081 | 0.041 | 1.987 | 0.0472* | 0.1011 |  |  |
| **Male 75-84 ADL** | | | | | | | | |
| Year | | 0.013 | 0.008 | 1.676 | 0.0939 | 0.1878 |  |  |
