# Supplementary material for: Age-specific trends in limitations of daily activities in American adults aged 50–84 by race and ethnicity, 2000–2018
Source: PLoS One. 2026 Feb 23;21(2):e0340694. doi: 10.1371/journal.pone.0340694 (PMC12928396; doi:10.1371/journal.pone.0340694)
Supplement: S5 Table — (DOCX) [file pone.0340694.s005.docx]

**Table 5S.** Maximum Variance Inflation Factor (VIF) testing for multicollinearity in models 1-12. Note: A VIF > 5 suggests potential multicollinearity.

| **Model** | **Term** | **Max Variance Inflation Factor** |  |  |
| --- | --- | --- | --- | --- |
| **Male 50-64 ADL** | | 2.53 |  |  |
| **Male 65-74 ADL** | | 2.86 |  |  |
| **Male 75-84 ADL** | | 2.61 |  |  |
| **Female 50-64 ADL** | | 2.81 |  |  |
| **Female 65-74 ADL** | | 3.07 |  |  |
| **Female 75-84 ADL** | | 2.55 |  |  |
| **Male 50-64 IADL** | | 2.61 |  |  |
| **Male 65-74 IADL** | | 2.69 |  |  |
| **Male 75-84 IADL** | | 2.43 |  |  |
| **Female 50-64 IADL** | | 2.71 |  |  |
| **Female 65-74 IADL** | | 2.68 |  |  |
| **Female 75-84 IADL** | | 2.49 |  |  |
