# Supplementary material for: Age-specific trends in limitations of daily activities in American adults aged 50–84 by race and ethnicity, 2000–2018
Source: PLoS One. 2026 Feb 23;21(2):e0340694. doi: 10.1371/journal.pone.0340694 (PMC12928396; doi:10.1371/journal.pone.0340694)
Supplement: S6 Table — (DOCX) [file pone.0340694.s006.docx]

**Table 6S.** Post-hoc power analyses of proportions across groups by sex, age, and race-ethnicity

Note: power calculated using two-sample test of proportions (significance with <.05)

| **Race/Ethnicity-Age** | **Sample Sizes (2000-09 / 2010-18)** | **ADL Change** | **ADL Power (%)** | **IADL Change** | **IADL Power (%)** |
| --- | --- | --- | --- | --- | --- |
| **Female** | | | | | |
| NH-White 50-64 | 48,156 / 59,228 | 1.51% → 1.93% | 100.0 | 3.42% → 3.95% | 99.6 |
| NH-White 65-74 | 21,400 / 27,347 | 2.85% → 2.99% | 14.9 | 5.83% → 5.5% | 34.6 |
| NH-White 75-84 | 14,045 / 17,341 | 6.42% → 6.43% | 5.0 | 13.61% → 12.09% | 97.9 |
| NH-Black 50-64 | 9,366 / 11,823 | 3.26% → 3.86% | 64.9 | 6.53% → 6.99% | 26.3 |
| NH-Black 65-74 | 3,674 / 4,187 | 5.95% → 6.5% | 17.2 | 11.1% → 10.56% | 12.0 |
| NH-Black 75-84 | 1,873 / 2,354 | 12.27% → 13.18% | 14.3 | 23.38% → 20.5% | 61.4 |
| Hispanic US Born 50-64 | 4,031 / 4,594 | 2.73% → 3.75% | 76.3 | 5.63% → 6.61% | 47.5 |
| Hispanic US Born 65-74 | 1,624 / 1,555 | 5.58% → 5.74% | 5.4 | 9.96% → 10.56% | 8.6 |
| Hispanic US Born 75-84 | 809 / 902 | 12.1% → 12.02% | 5.0 | 20.91% → 18.28% | 27.8 |
| Hispanic Foreign Born 50-64 | 6,330 / 7,844 | 1.5% → 1.58% | 6.7 | 2.57% → 2.82% | 15.0 |
| Hispanic Foreign Born 65-74 | 2,127 / 2,114 | 4.31% → 4.79% | 11.7 | 7.09% → 7.89% | 16.8 |
| Hispanic Foreign Born 75-84 | 994 / 1,185 | 11.51% → 13.58% | 30.7 | 17.24% → 18.6% | 13.1 |
| **Male** | | | | | |
| NH-White 50-64 | 43,874 / 53,961 | 1.51% → 1.93% | 99.9 | 3.42% → 3.95% | 99.2 |
| NH-White 65-74 | 18,457 / 23,586 | 2.85% → 2.99% | 13.5 | 5.83% → 5.5% | 30.6 |
| NH-White 75-84 | 10,234 / 12,636 | 6.42% → 6.43% | 5.0 | 13.61% → 12.09% | 92.7 |
| NH-White 50-64 | 43,874 / 53,961 | 1.51% → 1.93% | 99.9 | 3.42% → 3.95% | 99.2 |
| NH-White 65-74 | 18,457 / 23,586 | 2.85% → 2.99% | 13.5 | 5.83% → 5.5% | 30.6 |
| NH-White 75-84 | 10,234 / 12,636 | 6.42% → 6.43% | 5.0 | 13.61% → 12.09% | 92.7 |
| Hispanic US Born 50-64 | 3,672 / 4,185 | 2.73% → 3.75% | 72.4 | 5.63% → 6.61% | 44.0 |
| Hispanic US Born 65-74 | 1,400 / 1,341 | 5.58% → 5.74% | 5.4 | 9.96% → 10.56% | 8.1 |
| Hispanic US Born 75-84 | 590 / 657 | 12.1% → 12.02% | 5.0 | 20.91% → 18.28% | 21.5 |
| Hispanic Foreign Born 50-64 | 5,767 / 7,147 | 1.5% → 1.58% | 6.6 | 2.57% → 2.82% | 14.1 |
| Hispanic Foreign Born 65-74 | 1,834 / 1,823 | 4.31% → 4.79% | 10.7 | 7.09% → 7.89% | 15.1 |
| Hispanic Foreign Born 75-84 | 724 / 864 | 11.51% → 13.58% | 23.7 | 17.24% → 18.6% | 10.8 |
