# Supplementary material for: Age-specific trends in limitations of daily activities in American adults aged 50–84 by race and ethnicity, 2000–2018
Source: PLoS One. 2026 Feb 23;21(2):e0340694. doi: 10.1371/journal.pone.0340694 (PMC12928396; doi:10.1371/journal.pone.0340694)
Supplement: S7 Table — (DOCX) [file pone.0340694.s007.docx]

**Table 7S.** Names and values for key variables in the analyses

Note: Codes shown are original NHIS values. Race/ethnicity was recoded into four categories: NH-White, NH-Black, Hispanic US Born, and Hispanic Foreign Born. Education was recoded into three categories for analysis. Additional details are in the OSF repository

| **Variable** | **Code** | **Label** |
| --- | --- | --- |
| **SEX** | | |
|  | 1 | Male |
|  | 2 | Female |
| **RACENEW (Race)** | | |
|  | 100 | White only |
|  | 200 | Black/African American only |
|  | 300 | American Indian/Alaska Native only |
|  | 400 | Asian only |
|  | 500+ | Other/Multiple Race |
| **HISPYN (Hispanic Ethnicity)** | | |
|  | 1 | Not Hispanic |
|  | 2 | Hispanic |
| **USBORN (Nativity)** | | |
|  | 10-12 | No, born outside U.S. |
|  | 20 | Yes, born in U.S. |
|  | 96+ | Unknown/NIU |
| **EDUC (Education)** | | |
|  | 000-116 | < High school |
|  | 200-202 | High school/GED |
|  | 300-303 | Some college/AA |
|  | 400 | Bachelor's degree (BA) |
|  | 500-522 | Graduate degree |
|  | 996+ | Unknown |
| **LADL (ADL Limitations)** | | |
|  | 1 | No limitation |
|  | 2 | Has ADL limitation |
| **LAIADL (IADL Limitations)** | | |
|  | 1 | No limitation |
|  | 2 | Has IADL limitation |
| **LAWALK (Difficulty walking without equipment) – for sensitivity analyses** | | |
|  | 1 | No |
|  | 2 | Yes |
| **LAMEMRY (Activities limited by difficulty remembering) – for sensitivity analyses** | | |
|  | 1 | No |
|  | 2 | Yes |
