# Supplementary material for: Age-specific trends in limitations of daily activities in American adults aged 50–84 by race and ethnicity, 2000–2018
Source: PLoS One. 2026 Feb 23;21(2):e0340694. doi: 10.1371/journal.pone.0340694 (PMC12928396; doi:10.1371/journal.pone.0340694)
Supplement: Attachment — Submitted filename: Peer Review PONE - D - 25 - 30426.pdf [file pone.0340694.s020.pdf]

Introduction

1. Recommend not starting the first sentence with “After”
2. Recommend rephrasing the line “another important story”
3. Disability has been used numerous times during the introduction however this was not defined clearly. Needs earlier definition as most of the understanding surrounding the introduction revolves on the reader having a clear idea and concept of disability.

Overview:
The reader does not have clear definitions and understandings of disability and limitations therefore following along the ideas that are being presented remains ambiguous.
It would be helpful to put in hard factual numerical data to guide the reader as to the current and or past understanding regarding disability to give a frame of reference.
The majority of the introduction remains speculation on previous studies and pointing out unclear aspects of literature however without context the importance of these discrepancies have minimal impact on the reader
The overall pace and macrostructure of the introduction remains vague and does not guide the reader through specific points adequately and easily

Statistical Analysis

1. No power calculation shown.
2. What calculation was done to ensure the model was well fitted vs overfitted
3. No sensitivity analysis was noted
4. No correction for multiple comparisons was mentioned e.g Bonferroni
5. How was missing data managed
6. No notation for test of multicollinearity
7. No external validation or bootstrapping mentioned
8. Data on the software information needs to be listed

Overview:
Deficit noted regarding the robustness of the statistical analysis to cement the clarity of the information.

Results

1. What is BA? This acronym has not been defined however is utilized in the Tables
2. Table 2, 3 and 4 needs better formatting. Recommend dividing table and having the 95% confidence interval as a separate column. Very busy and over populated
3. Would be helpful to have the information compared within ethnicity along the age groups to see a trend among the ethnicity in one format eg. Graph or Bar chart

Overview:
This data can be better represented in formats that highlight trends especially in the distinct groups. Table format can still be utilized however visual format of the data would help increase the strength of the information represented

Discussion
1. Helpful to add the factual data in the 1980s and 1990s to help place the current numerical data in context from this study
2. Grammatical error for “ is still is the age group…” in 2nd paragraph
3. Helpful to add some data on the factors that have “ co - occurred with rising disability”
4. How can you explain the with the NH Black population
5. Limitations of the study is missing
6. Further exploration and utilization of the data is needed to be mentioned as well
7. How can this data be helpful from a clinical perspective?

Recommendation
- Major Revision
