# Supplementary material for: Age-specific trends in limitations of daily activities in American adults aged 50–84 by race and ethnicity, 2000–2018
Source: PLoS One. 2026 Feb 23;21(2):e0340694. doi: 10.1371/journal.pone.0340694 (PMC12928396; doi:10.1371/journal.pone.0340694)
Supplement: Attachment — Submitted filename: Review Report.docx [file pone.0340694.s021.docx]

Review Report

Manuscript Title: *Age-specific Trends in Limitations of Daily Activities in American Adults aged 50–84 by Race and Ethnicity, 2000–2010 and 2010–2018*
Journal: PLOS ONE

Summary

This article examines trends in limitations in Activities of Daily Living (ADLs) and Instrumental Activities of Daily Living (IADLs) among U.S. adults aged 50–84, with a focus on differences by sex, age group, and race/ethnicity. Using NHIS data (2000–2018) harmonized in IPUMS, the authors apply logistic regression with interaction terms to model prevalence trends, compare two decades (2000–2009 vs. 2010–2018), and report relative risks and predicted prevalences. The findings show that adverse disability trends observed in the 2000s persisted into 2010–2018, with the strongest increases in adults aged 50–64, and with marked deterioration among Hispanic men and women in the later decade.

Strengths

- Clear research focus: Appropriate distinction between two decades and between age groups.
- Robust data source: NHIS/IPUMS provides large, representative samples.
- Public health relevance: Addresses important disparities by race/ethnicity and age, with implications for health and social policy.

Major Concerns

1. Survey design and estimation

It is not clear whether the authors fully accounted for the NHIS complex survey design (weights, strata, PSU). Using only weights without design-based variance estimation can bias confidence intervals and p-values. Please describe in detail how survey design was incorporated and how marginal prevalences and contrasts were calculated.

2. Trend specification

The piecewise linear trend with a break in 2010 is reasonable, but the rationale needs elaboration. Why 2010?

3. Covariates and interpretation

- Age range 50–84: Please justify the exclusion of ≥85 (top-coded in NHIS). Consider sensitivity analyses with 70–84 as a separate grouping.
- Education: Since rising educational attainment partly explains earlier disability declines, controlling for education may introduce over-adjustment. Please show results with and without education as a covariate.
- Nativity: As nativity is included, an interaction with race/ethnicity should be considered, since Hispanic population composition shifted greatly in this period. At minimum, report descriptives of nativity within racial/ethnic groups.
- Missing data: Please report the extent and handling (listwise deletion vs. imputation).

4. ADL/IADL measurement

Please provide a mapping of NHIS/IPUMS variables used to construct ADL and IADL measures, and confirm comparability across years. A supplementary table listing items, years available, and coding is recommended. Consider an alternative definition (≥2 limitations) as a robustness check.

5. Reporting of effect sizes

Relative risks derived from marginal predictions are appropriate, but absolute differences in prevalence (percentage points) are more interpretable for public health. Please report both.

6. Multiple testing

Given the numerous stratifications (age × sex × race/ethnicity × outcome), please address multiplicity. Either apply false discovery rate adjustment or explicitly frame analyses as exploratory and emphasize effect sizes and confidence intervals.

7. Supplementary material inconsistencies

There are several errors in supplementary tables:

- Table 6A (ADL, women 75–84): coefficient 0.068, SE 0.007 → reported p = 0.380 is inconsistent (z ≈ 9.7, p < .001).
- Table 8A (IADL, men 65–74): “Low Education = 0.934 0.934 <0.01**” — SE is duplicated; likely a typo.
- Table 9A (IADL, men 75–84): coefficient 0.117, SE 0.024, but p = 0.625 (should be highly significant).
- Decimal punctuation inconsistent (comma vs. point).

Please regenerate all tables directly from code to avoid transcription errors.

8. Suggested sensitivity analyses

- Survey vs. non-survey estimation (to show effect of weights/design).
- Interaction nativity × race/ethnicity.
- Stricter ADL/IADL definitions (≥2 limitations).

Optional: link to external indicators (e.g., opioid epidemic, comorbidity prevalence) for ecological robustness.

Minor Concerns

- Terminology: Use consistent forms “NH White,” “NH Black,” “Hispanic/Latino.”
- Language/grammar: Correct double semicolons, harmonize capitalization, and shorten overly long sentences.
- References: Standardize to PLOS ONE style; include all DOIs; check duplicates (e.g., Martin & Schoeni 2014 appears twice).
- Figures: Include one main figure (ages 50–64; ADL and IADL by race/ethnicity). Leave full annual trend figures in supplement.
- Data availability: Provide IPUMS extract ID, date of download, and repository with analysis code (Zenodo/OSF/GitHub).

Validity of Conclusions

The main findings - persistent adverse trends, disproportionate burden among middle-aged adults, and widening disparities particularly affecting Hispanics post-2010 are plausible and consistent with prior literature. However, the issues with survey design, trend specification, and supplementary table errors must be addressed before publication.

Recommendation

Major Revision.
The study addresses an important public health question with high-quality data. With correction of statistical methods, improved reporting, and cleaned supplementary material, the manuscript will be suitable for publication in PLOS ONE.
