# Supplementary material for: Age-specific trends in limitations of daily activities in American adults aged 50–84 by race and ethnicity, 2000–2018
Source: PLoS One. 2026 Feb 23;21(2):e0340694. doi: 10.1371/journal.pone.0340694 (PMC12928396; doi:10.1371/journal.pone.0340694)
Supplement: Attachment — Submitted filename: Response to Reviewers PONE.docx [file pone.0340694.s022.docx]

*We thank each reviewer for their careful assessment of our manuscript and for their suggested improvements. Below, we outline the changes we have made to the made to the manuscript.*

**Reviewer 1:**

Introduction

**Comment**

Recommend not starting the first sentence with “After”

**Response**

We have edited the first sentence accordingly.

**Comment**

Recommend rephrasing the line “another important story”

**Response**

We have edited the sentence accordingly.

**Comment**

The reviewer provided several comments around defining disability and limitations:

Disability has been used numerous times during the introduction however this was not defined clearly.

Needs earlier definition as most of the understanding surrounding the introduction revolves on the reader having a clear idea and concept of disability.

The reader does not have clear definitions and understandings of disability and limitations therefore following along the ideas that are being presented remains ambiguous. It would be helpful to put in hard factual numerical data to guide the reader as to the current and or past understanding regarding disability to give a frame of reference.

**Response**

We added the following paragraphs in the introduction section, expanding on the disability measures, concepts and previous studies.

“Limitations in activities of daily living (ADLs) and instrumental activities of daily living (IADLs) have long been considered gold standard measures for assessing functional disability in population-based research (Katz, 1963, 1983; Lawton & Brody, 1969). These measures capture fundamental aspects of independent functioning—from basic self-care tasks like bathing and dressing (ADLs) to more complex activities like managing finances and medications (IADLs). These measures are often used in the context of the disablement process (Verbrugge & Jette, 1994), a framework that demonstrates how chronic conditions, injuries, and impairments lead to functional limitations that hinder quality of life and the ability to perform daily activities (Verbrugge et al., 1989; Zajacova & Margolis, 2025). Over the 1980s and 1990s, the U.S. experienced sizeable declines in national-level disability prevalence as measured by ADLs and IADLs (Freedman et al., 2002; Martin et al., 2010; Schoeni et al., 2001; Waidmann & Liu, 2000). Various explanations for this improvement have been identified including increases in educational attainment (Freedman et al., 2002; Martin et al., 2010; Schoeni et al., 2001), improvements in medical care, and the long-term benefits of improvements in earlier-in-life health (Freedman & Martin, 1998). The first decade of the 2000s brought about a notable shift in U.S. disability trends measured by these indicators. Previous studies that analyzed this period found that the declining ADL and IADL prevalence among Americans aged 55 and older stagnated (Freedman et al., 2013; Lakdawalla et al., 2004; Seeman et al., 2010; Tsai, 2017). Some other studies suggest that disability increased among middle-aged individuals, reaching by 2010 a 1.7% prevalence of ADL and 4% of IADL for those aged between 40 and 65, and above near 9% and 12% respectively for individuals aged 65+ (Freedman et al., 2013; Martin, Freedman, et al., 2010; Martin & Schoeni, 2014). Other group classifications presented similar trends: among those aged 55-69, IADL limitations increased by an average of 1.33% annually between 1998-2012, with most of the increase concentrated toward the end of the period (Choi et al., 2016). “

**Comment**

The majority of the introduction remains speculation on previous studies and pointing out unclear aspects of literature however without context the importance of these discrepancies have minimal impact on the reader

The overall pace and macrostructure of the introduction remains vague and does not guide the reader through specific points adequately and easily

**Response**

We have re-structured the Introduction to better motivate the analysis. As noted above, we provide a more detailed and careful review of the existing literature on national disability trends. We then include a dedicated paragraph on why studying disability trends in the post-2010 period is important, starting in page 2 with this paragraph:

Trends after 2010 remain understudied. Around 2010, U.S. life expectancy began to stagnate and even decline driven by stagnating declines in cardiovascular disease mortality and rising mortality from drug overdoses, suicides, and alcoholic liver disease. Broader deteriorations in population health have also been documented after 2010 (Case & Deaton, 2015, 2017). The 2008 economic crisis and its aftermath provide additional motivation for examining the post-2010 period. Economic recessions have been shown to have lasting effects on population health, particularly through increased financial strain, job loss, reduced access to healthcare, and heightened psychological distress (Burgard & Kalousova, 2015; Catalano et al., 2011). It remains unclear whether this event is also associated with a turning point for disability trends. These effects may be particularly pronounced for middle-aged adults approaching retirement who experienced job displacement during peak earning years. Moreover, the post-2010 period saw accelerating increases in obesity levels and the continued escalation of the opioid epidemic, which are key risk factors for functional limitations (Alley & Chang, 2007; Stokes et al., 2020; Zajacova et al., 2021).

Statistical Analysis

1. No power calculation shown.

It's unclear which kind of power analysis is requested here, but to comply with this suggestion, we conducted post-hoc power analyses (Table 6S in the supplementary material) to assess our ability to detect changes in disability prevalence between the two decades (2000-2009 vs 2010-2018) using two-sample tests of proportions (significance lower than 0.05). Our primary findings regarding disability trends among middle-aged adults (50-64) were well-powered for Non-Hispanic Whites, Non-Hispanic Blacks and US-Born Hispanics. Statistical power was more limited for older age groups (65-74 and 75-84) and smaller demographic subgroups.

However, we note that power analyses are more commonly applied to hypothesis-testing studies designed to detect specific effect sizes, rather than to descriptive epidemiological studies documenting population-level trends as this one. Our study's primary objective is to describe and characterize temporal patterns in disability prevalence across demographic groups, not to test a priori hypotheses about specific differences. The substantial sample sizes from the NHIS provide sufficient precision to detect meaningful population-level changes, as evidenced by the narrow confidence intervals around our prevalence estimates. Moreover, similar descriptive trend analyses in the disability literature cited in the paper (i.e: Choi et al., 2016; Freedman et al., 2013; Martin, Freedman, et al., 2010) have not required formal power calculations, as the goal is comprehensive description rather than hypothesis confirmation.

2. What calculation was done to ensure the model was well fitted vs overfitted ????

We now provide model fit estimates based on R-squared values (Supplementary Material, Table S3). We computed three types of R-squared (McFadden, Nagelkerke, and Pseudo R-squared), which all yielded similar values ranging from 0.018 to 0.047 across models. While these values appear modest, we believe they are appropriate and expected for this type of analysis. Our models intentionally include only key demographic covariates (age, race/ethnicity, education, and time period) because our goal is to describe population-level epidemiological trends rather than to build predictive models or explain individual-level variation in disability. In large population-based studies examining broad temporal patterns, low R-squared values are typical and do not indicate poor model performance. The precision of our estimates is reflected in the narrow confidence intervals rather than in R-squared values. Furthermore, our parsimonious modeling approach focuses on main effects and key interactions guards against overfitting. The consistency of findings across different model specifications and the social plausibility of the observed trends provide additional confidence in our results.

3. No sensitivity analysis was noted

We now provide several sensitivity analyses, which are presented in the supplementary material, from figures 4S to 12S, analyzing the age-specific predicted probabilities of the following:

-Models without survey weights to illustrate the effect of the complex survey design. The results remained consistent with our main findings.

-We tested models without education to assess its influence. Results were largely similar across groups, with the exception of the Hispanic foreign-born subgroup, which showed some variation.

-Due to limitations in the data (i.e., individual ADL/IADL items were not asked separately but altogether in a single question), we used proxy measures. Specifically, we used difficulty walking without equipment as a stand-in for ADL limitations, and presence of memory problems (if the respondent had activities limited by difficulty remembering things) as a proxy for IADL limitations. While these are imperfect substitutes, they capture related functional impairments. We acknowledge that stricter definitions (e.g., ≥2 limitations) would be ideal but the questions asked are not exactly the ones that make the ADL/IADL definitions, and we are open to exploring additional proxies available in the dataset if the reviewer deems it necessary.

Across all sensitivity analyses, predicted probabilities remained consistent with our main estimates, supporting the robustness of our findings.

4. No correction for multiple comparisons was mentioned e.g Bonferroni

We used False Discovery Rate (FDR) adjustment, using the Benjamini-Hochberg procedure, available in Table 4S of the supplementary material. Only one term that originally had a p value lower than 0.05 exceeded that threshold, but it does not imply a significant trend change either way.

5. How was missing data managed

We changed the methods section and described which observations were removed in order to work with a complete case-analysis. Results are Observations on missing data are Table 1S of the supplementary material

6. No notation for test of multicollinearity

We now are showing tests for multicollinearity in Table 5S of the supplementary material. In all cases the Variance Inflation Factor (VIF) results were under 3 (which is under the rule of thumb threshold value of 5 to consider that there might be multicollinearity among variables)

7. No external validation or bootstrapping mentioned

We did not perform external validation as our primary goal was to describe trends at a population level using standard epidemiological techniques, not building a predictive model of disability. Our methods and results seem to be consistent with related studies that we mention in the discussion.

8. Data on the software information needs to be listed

We clarified now the software in the methos section. We used R software, version 4.3.1 in the methods sections and specially the *survey* package, that we used for our estimations.

Overview: Deficit noted regarding the robustness of the statistical analysis to cement the clarity of the information.

Thank you for your suggestions We agree with the reviewer and we changed our analysis accordingly as shown.

Results

What is BA? This acronym has not been defined however is utilized in the Tables 2. Table 2, 3 and 4 needs better formatting.

Recommend dividing table and having the 95% confidence interval as a separate column. Very busy and over populated

3. Would be helpful to have the information compared within ethnicity along the age groups to see a trend among the ethnicity in one format eg. Graph or Bar chart

Overview: This data can be better represented in formats that highlight trends especially in the distinct groups.

Table format can still be utilized however visual format of the data would help increase the strength of the information represented Discussion

Thank you for your suggestion. We agree with the reviewer. This was clarified (Bachelor’s Degree and Beyond). Also, we redid the tables and presented figures instead (Figures 1 and 2) because we believe is easier to understand, but showing the same results as the tables before.

1. Helpful to add the factual data in the 1980s and 1990s to help place the current numerical data in context from this study

2. Grammatical error for “ is still is the age group…” in 2nd paragraph

3. Helpful to add some data on the factors that have “ co - occurred with rising disability”

We modified the grammar and added a mention of a Freedman et al. (2004) study quantifying the declining prevalence trend in the 1990 for ADL and IADL among Americans.

4. How can you explain the with the NH Black population

There might be something missing here with the reviewer’s comment, so we were unable to address this.

5. Limitations of the study is missing

We added the following section in the discussion, near the end of the manuscript:

This study has several limitations that should be considered when interpreting our findings. First, the NHIS relies on self-reported data, which may be subject to reporting bias and influenced by respondents' understanding of disability questions, cultural interpretations of functional limitations, and language barriers, particularly among foreign-born populations. Second, our analysis is descriptive in nature and does not employ a causal framework; therefore, we cannot identify the mechanisms driving the observed trends or establish causal relationships between demographic characteristics and disability changes. Third, our parsimonious modeling approach intentionally focused on demographic covariates (age, race/ethnicity, education and time period) to clearly describe population-level trends, but this means we did not account for other potentially important factors such as wealth, chronic disease burden, occupational exposures, or healthcare access that may contribute to or confound the observed patterns. Fourth, sample sizes were limited for certain demographic subgroups, and we could not consider other ethnic groups. Despite these limitations, the large, nationally representative sample and consistent data collection methods across nearly two decades provide valuable insights into evolving disability patterns across diverse demographic groups in the United States.

6. Further exploration and utilization of the data is needed to be mentioned as well

7. How can this data be helpful from a clinical perspective? Recommendation - Major Revision

This is a comment that makes us think a bit more about our findings, thanks. We believe the most important thing about this paper are the substantial increases in disability among middle-aged adults (ages 50-64) have important clinical implications. This pre-retirement age group experienced the largest relative increases in functional limitations, with some subgroups—particularly US-born Hispanic women—experiencing larger increases in ADL limitations between 2000 and 2018. From a clinical perspective, these findings highlight the need for earlier screening and preventive interventions targeting functional decline in patients in their 50s and early 60s, and this is the trend among all race/ethnicity groups, but with changes in intensity. Additionally, clinicians and geriartricians should be aware that the traditional focus on disability management in adults aged 65+ may need to shift earlier in the life course, as functional limitations are increasingly manifesting during the working years when individuals may still be employed and lack access to Medicare. Early identification and intervention during middle age, when functional decline may be more modifiable, could help prevent or delay more severe disability in later life and reduce the burden on both individuals and the healthcare system.

Thanks to the reviewer for its input and help. We really believe the manuscript has improved because of that.

**Reviewer 2:**

Summary

This article examines trends in limitations in Activities of Daily Living (ADLs) and Instrumental Activities of Daily Living (IADLs) among U.S. adults aged 50–84, with a focus on differences by sex, age group, and race/ethnicity. Using NHIS data (2000–2018) harmonized in IPUMS, the authors apply logistic regression with interaction terms to model prevalence trends, compare two decades (2000–2009 vs. 2010–2018), and report relative risks and predicted prevalences. The findings show that adverse disability trends observed in the 2000s persisted into 2010–2018, with the strongest increases in adults aged 50–64, and with marked deterioration among Hispanic men and women in the later decade.

Thank you very much for your review. We found it very helpful. In color blue are our responses.

Strengths

- Clear research focus: Appropriate distinction between two decades and between age groups.
- Robust data source: NHIS/IPUMS provides large, representative samples.
- Public health relevance: Addresses important disparities by race/ethnicity and age, with implications for health and social policy.

Major Concerns

1. Survey design and estimation

It is not clear whether the authors fully accounted for the NHIS complex survey design (weights, strata, PSU). Using only weights without design-based variance estimation can bias confidence intervals and p-values. Please describe in detail how survey design was incorporated and how marginal prevalences and contrasts were calculated.

Thank you for your comment. You are correct, we did not consider the PSU and strata, just the person weights. We acknowledged this at the end of the methods section, recalculated everything considering the multi-layered survey design weights (any difference with just using the person weights was observed in variance estimation, not in the point estimates) and also, we presented an unweighted estimation as a sensitivity analysis (Available in Figures 10S to 12S in the supplementary material) as suggested by the reviewer.

Sensitivity analyses presented in the supplementary material include age-specific predicted probabilities of the prevalence trends analysis of the without sampling (Figures 4S-6S), models without controlling by education (Figures 7S-9S), and replications of the analyses with difficulties in walking without especial equipment as a proxy for ADL and having any memory troubles to do activities as a proxy for IADL, available in Figures 10S to 12S).

2. Trend specification

The piecewise linear trend with a break in 2010 is reasonable, but the rationale needs elaboration. Why 2010?

We acknowledge that the choice of 2010 as the inflection point in our piecewise linear trend is somewhat arbitrary. Rather than applying multiple spline terms or group-specific joinpoint regressions—which could complicate interpretation and reduce comparability across racial/ethnic groups—we opted for a single, consistent break point for all groups.

The decision to use 2010 was guided by substantive knowledge on population health trends rather than data-driven model selection. As elaborated in the revised introduction, the post-2010 period marks a notable shift in U.S. population health trends, and the aftermath of the 2008 economic crisis. These developments suggest a plausible turning point in disability trends, particularly among vulnerable populations. While we recognize that other break points could be justified, we believe 2010 represents a reasonable choice based on broader contextual factors rather than statistical optimization. The introduction section now reads:

“Trends after 2010 remain understudied. Around 2010, U.S. life expectancy began to stagnate and even decline driven by stagnating declines in cardiovascular disease mortality and rising mortality from drug overdoses, suicides, and alcoholic liver disease. Broader deteriorations in population health have also been documented after 2010 (Case & Deaton, 2015, 2017). The 2008 economic crisis and its aftermath provide additional motivation for examining the post-2010 period. Economic recessions have been shown to have lasting effects on population health, particularly through increased financial strain, job loss, reduced access to healthcare, and heightened psychological distress (Burgard & Kalousova, 2015; Catalano et al., 2011). It remains unclear whether this event is also associated with a turning point for disability trends. These effects may be particularly pronounced for middle-aged adults approaching retirement who experienced job displacement during peak earning years. Moreover, the post-2010 period saw accelerating increases in obesity levels and the continued escalation of the opioid epidemic, which are key risk factors for functional limitations (Alley & Chang, 2007; Stokes et al., 2020; Zajacova et al., 2021).”

3. Covariates and interpretation

- Age range 50–84: Please justify the exclusion of ≥85 (top-coded in NHIS). Consider sensitivity analyses with 70–84 as a separate grouping.
- Education: Since rising educational attainment partly explains earlier disability declines, controlling for education may introduce over-adjustment. Please show results with and without education as a covariate.
- Nativity: As nativity is included, an interaction with race/ethnicity should be considered, since Hispanic population composition shifted greatly in this period. At minimum, report descriptives of nativity within racial/ethnic groups.
- Missing data: Please report the extent and handling (listwise deletion vs. imputation).

Thanks for the suggestions. A lot of good points here!. We’ll go point by point.

**Nativity**: This was a very good reccomendation. Upon review, we found that a substantial proportion of the Hispanic population was foreign-born, so interactions had to consider this. However, since we are talking about a substantive portion of Hispanic population, close to half of it (and this number is consistent across both decades), we therefore separated U.S.-born and foreign-born Hispanics in our main models to better capture this heterogeneity. To avoid redundancy and potential collinearity, we did not include nativity for non-Hispanic groups in the models, as the foreign-born share among them was below 10% (and when we tried, it was non-significant probably due to sample sizes). Descriptive statistics on nativity by race/ethnicity are provided in Table 1S of the supplementary material.

**Age range (50–84)**: We chose to focus on adults aged 50–84 for two main reasons. First, the 50–64 age group is particularly relevant given its worsening health trends and vulnerability due to being below Medicare eligibility age. Second, we wanted to preserve granularity in older age groups by separately analyzing ages 65–74 and 75–84, as combining them could obscure divergent trends (and visual inspection from figures 1S to 3S indicates that visual patterns remain similar in any case). While we acknowledge that other studies have used different age specifications, we believe this grouping best serves our analysis.

We also excluded individuals aged 85 and older due to NHIS public-use data top-coding at age 85, which prevents us from assessing changes in age distribution or controlling for single-year age variation in this group. This rationale has been added to the methods section.

**Education**: We conducted sensitivity analyses excluding education as a covariate to assess potential over-adjustment, given its role in explaining earlier disability declines (Figures 7S to 9S in the supplementary material). The predicted probabilities remained largely consistent across groups, with some variation observed for the Hispanic foreign-born subgroup. These results are included in the supplementary material.

**Missing data**: We removed 1,697 cases due to missing nativity information, which represented only 0.35% of the sample. We opted for complete case analysis, as the small proportion of missing data was unlikely to bias our estimates. This approach is now described in the methods section, and missingness details are provided in Table 1S.

4. ADL/IADL measurement

Please provide a mapping of NHIS/IPUMS variables used to construct ADL and IADL measures, and confirm comparability across years. A supplementary table listing items, years available, and coding is recommended. Consider an alternative definition (≥2 limitations) as a robustness check.

Thanks for the suggestion. We are attaching the IPUMS codebook of the main variables used in the supplementary material (Table 7S) and in the open source framework with the code.

5. Reporting of effect sizes

Relative risks derived from marginal predictions are appropriate, but absolute differences in prevalence (percentage points) are more interpretable for public health. Please report both.

Figures 6 to 8 now report prevalence changes, so we can see them across decades. We incorporated them in the analysis among the risk ratios

6. Multiple testing

Given the numerous stratifications (age × sex × race/ethnicity × outcome), please address multiplicity. Either apply false discovery rate adjustment or explicitly frame analyses as exploratory and emphasize effect sizes and confidence intervals.

Thank you for your comment. We used False Discovery Rate (FDR) adjustment, using the Benjamini-Hochberg procedure. Only one term that originally had a p value lower than 0.05 exceeded that threshold, but it does not imply a significant trend change either way. Table 4S in the supplementary material presents the results.

7. Supplementary material inconsistencies

There are several errors in supplementary tables:

- Table 6A (ADL, women 75–84): coefficient 0.068, SE 0.007 → reported p = 0.380 is inconsistent (z ≈ 9.7, p < .001).
- Table 8A (IADL, men 65–74): “Low Education = 0.934 0.934 <0.01**” — SE is duplicated; likely a typo.
- Table 9A (IADL, men 75–84): coefficient 0.117, SE 0.024, but p = 0.625 (should be highly significant).
- Decimal punctuation inconsistent (comma vs. point).

Please regenerate all tables directly from code to avoid transcription errors.

We did again all tables, and in Table 2S in the supplementary material (and all of them) we have the coefficient of the main models, copied straight from the code as requested.

8. Suggested sensitivity analyses

- Survey vs. non-survey estimation (to show effect of weights/design).
- Interaction nativity × race/ethnicity.
- Stricter ADL/IADL definitions (≥2 limitations).

Optional: link to external indicators (e.g., opioid epidemic, comorbidity prevalence) for ecological robustness.

As we mentioned before, we divided the Hispanic group in two (US-Born and Foreign-Born) since it’s evenly split and we believe that’s a good contribution based on the reviewer suggestion. Besides that, we presented the following sensitivity analyses in the supplementary material:

In addition to this, we conducted several sensitivity analyses, which are presented in the supplementary material, from figures 4S to 12S, analyzing the age-specific predicted probabilities of the following:

Models without survey weights to illustrate the effect of the complex survey design. The results remained consistent with our main findings.

We tested models without education to assess its influence. Results were largely similar across groups, with the exception of the Hispanic foreign-born subgroup, which showed some variation.

Due to limitations in the data (i.e., individual ADL/IADL items were not asked separately but altogether in a single question), we used proxy measures. Specifically, we used difficulty walking without equipment as a stand-in for ADL limitations, and presence of memory problems (if the respondent had activities limited by difficulty remembering things) as a proxy for IADL limitations. While these are imperfect substitutes, they capture related functional impairments. We acknowledge that stricter definitions (e.g., ≥2 limitations) would be ideal but the questions asked are not exactly the ones that make the ADL/IADL definitions, and we are open to exploring additional proxies available in the dataset if the reviewer deems it necessary.

Across all sensitivity analyses, predicted probabilities remained consistent with our main estimates, supporting the robustness of our findings.

Minor Concerns

- Terminology: Use consistent forms “NH White,” “NH Black,” “Hispanic/Latino.”
- Language/grammar: Correct double semicolons, harmonize capitalization, and shorten overly long sentences.
- References: Standardize to PLOS ONE style; include all DOIs; check duplicates (e.g., Martin & Schoeni 2014 appears twice).
- Figures: Include one main figure (ages 50–64; ADL and IADL by race/ethnicity). Leave full annual trend figures in supplement.
- Data availability: Provide IPUMS extract ID, date of download, and repository with analysis code (Zenodo/OSF/GitHub).

We corrected terminology, improved grammar and checked references (dois were added if found).

Figures: We put the annual trends in the supplement, but we added prevalence estimates, risk ratios and change in the main text, since we believe they add value to the analysis based on the reviewers’ suggestions.

We now provide the OSF code with the main and complementary analyses (<https://osf>.io/6pfth/?view_only=f460587c0f91438b872812b403d51e49). We are not sure if we can add the IPUMS data without permission (and we are not sure where is the exact extract ID in the website), but we are happy to indicate which variables we used and its treatment as explained in Table S7 and the codebook is also available in the OSF. Last date of download is October 25th, 2025

Validity of Conclusions

The main findings - persistent adverse trends, disproportionate burden among middle-aged adults, and widening disparities particularly affecting Hispanics post-2010 are plausible and consistent with prior literature. However, the issues with survey design, trend specification, and supplementary table errors must be addressed before publication.

Thanks to the reviewer for its input and help. We really believe the manuscript has improved because of that.

Recommendation

Major Revision.
The study addresses an important public health question with high-quality data. With correction of statistical methods, improved reporting, and cleaned supplementary material, the manuscript will be suitable for publication in PLOS ONE.
